# Theta-burst stimulation causally affects side perception in the Deutsch’s octave illusion

**DOI:** 10.1038/s41598-018-31248-1

**Published:** 2018-08-27

**Authors:** Paolo Capotosto, Stefania della Penna, Vittorio Pizzella, Filippo Zappasodi, Gian Luca Romani, Risto J. Ilmoniemi, Alfredo Brancucci

**Affiliations:** 1 Department of Neuroscience, Imaging and Clinical Sciences, “G. d’Annunzio” University of Chieti and Pescara, Chieti, Italy; 2 ITAB-Institute of Advanced Biomedical Technologies, “G. d’Annunzio” University of Chieti and Pescara, Chieti, Italy; 3 Department of Neuroscience and Biomedical Engineering (NBE), Aalto University School of Science, Espoo, Finland; 4 Department of Psychological, Health and Territory Sciences, “G. d’Annunzio” University of Chieti and Pescara, Chieti, Italy

## Abstract

Deutsch’s octave illusion is produced by a sequence of two specular dichotic stimuli presented in alternation to the left and right ear causing an illusory segregation of pitch (frequency) and side (ear of origin). Previous studies have indicated that illusory perception of pitch takes place in temporo-frontal areas, whereas illusory perception of side is primarily associated to neural activity in parietal cortex and in particular in the inferior parietal lobule (IPL). Here we investigated the causal role of left IPL in the perception of side (ear of origin) during the octave illusion by following its inhibition through continuous theta-burst stimulation (cTBS), as compared to the left posterior intraparietal sulcus (pIPS), whose activity is thought to be unrelated to side perception during the illusion. We observed a prolonged modification in the side of the illusory perceived tone during the first 10 minutes following the stimulation. Specifically, while after cTBS over the left IPS subjects reported to perceive the last tone more often at the right compared to the left ear, cTBS over left IPL significantly reverted this distribution, as the number of last perceived tones at the right ear was smaller than at the left ear. Such alteration was not maintained in the successive 10 minutes. These results provide the first evidence of the causal involvement of the left IPL in the perception of side during the octave illusion.

## Introduction

**Figure 1:**
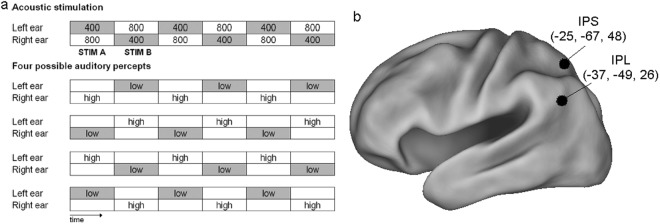
(**a**) Stimuli and percepts. Top, Acoustic stimulation sequence that elicits the Deutsch’s octave illusion (numbers indicate sine tone frequencies in hertz, each tone lasts 500 ms). Bottom, the four typical percepts arising during the listening to the upper stimulation in 99% of the population; “low” and “high” refer to the perceived pitch. Of note, outside from the context of the illusion, the 400-Hz tone would be perceived as the low tone and the 800-Hz one as the high tone. It can be noticed that both StimA and StimB can be perceived as low or high and at the left or right ear. (**b)** Inflated view of left hemisphere atlas brain with regions obtained from^7^ and^51^. Regions with Talairach coordinates (millimeters) are stimulated with cTBS in this experiment.

In the Deutsch’s octave illusion^1^, subjects perceive a high pitch in one ear and a low pitch in the other ear in a sequence of dichotically presented tones. It occurs when two sequences of tones alternating in frequency, e.g., between 400 and 800 Hz, are presented to the two ears so that when the left ear receives the 400-Hz tone, the right ear receives the 800-Hz tone, and vice versa (Fig. 1a). In these stimulation conditions, subjects typically perceive a single low-pitch tone at one ear alternating with a single high-pitch tone at the other ear. This percept contains two illusory elements. First, only one tone is perceived at a time, whereby throughout the stimulation two tones are presented at the same time, one at each ear. Second, one of the two tones (the low- or the high-pitch one) is perceived at the ear where it is not actually presented. Interestingly, right-handed listeners perceive the high tone in the right ear and the low tone in the left ear significantly more frequently than left-handed listeners^2^.

In the last years, the octave illusion has been proposed as one of the very few examples of perceptual bistability in the auditory domain^3–5^. This fact allows to investigate an intriguing aspect of neuroscience, i.e., the neural basis of consciousness, commonly addressed as the neural correlate of consciousness (NCC). In a series of previous studies, we^3,4,6–9^ and other research groups^10–12^ observed different aspects of the perceptual and neural activity underlying the Deutsch’s octave illusion. These studies have indicated that the areas playing a key role in the illusion are located beyond the primary auditory cortex. In particular, illusory perception of pitch has been shown to have a main neural counterpart bilaterally in Heschl’s gyrus, the superior and inferior frontal gyrus, and the insular cortex, whereas illusory perception of side is primarily associated to bilateral neural activity in the superior and middle frontal gyri, as well as in the inferior parietal lobule (IPL). Notably, the left IPL was also involved in the processing of the physical dichotic stimuli causing the illusory perception^7^. However, these findings do not directly reveal the role of these areas on perception, leaving the possibility of their activation being just an epiphenomenon. To test the causal role of one brain area on perception or behavior, cognitive neuroscience exploits two main methodologies: lesion studies^13^ and non-invasive brain stimulation that can produce “virtual lesions”^14^. These approaches share the idea that if one area of the brain is damaged, the behavior relying on this activity is impaired.

In the present study, we used repetitive transcranial magnetic stimulation (rTMS), specifically continuous theta-burst stimulation (cTBS), to inhibit the left IPL in order to test the possible causal role of this area in the deceived perception of side during the Deutsch’s octave illusion^8^. In addition, we inhibited the left inferior parietal sulcus (pIPS) as a control area. The hypothesis was that the ear in which the tone is perceived is altered only after the stimulation of the left IPL.

## Results

### Preliminary experiment

In the present experimental design, subjects were instructed to press one of two keyboard buttons describing the subjective properties of the last perceived tone at the end of each tone block (Fig. 1a). In particular they were asked to press “Q”, with left index finger, when the last tone was perceived at the left ear, and to press “P”, with the right index finger, when the last tone was perceived at the right ear. To test whether this paradigm is consistent with previous observations^8^ showing a right-ear preponderance (i.e., subjects more often perceived the last tone of the block at the right ear), we first enrolled a group of right-handed (Edinburgh Inventory Index^15^: 81 ± 13) subjects (*N* = 15; 5 females), who were asked to perform a run of the main experiment without cTBS. As expected, one-way ANOVA confirmed the previous findings (F1,14 = 10.42, *p* = 0.006), indicating that the subjects perceived the last tone of the block more often on the right (53.6% ± 4.3) than on the left (46.4% ± 4.3) ear.

### Main experiment

Fifteen right-handed volunteers (Edinburgh Inventory Index^15^: 84 ± 11) participated to the main experiment, and all of them performed two experimental session in which cTBS was delivered over left IPL or left IPS, respectively (Fig. 1b).

**Figure 2:**
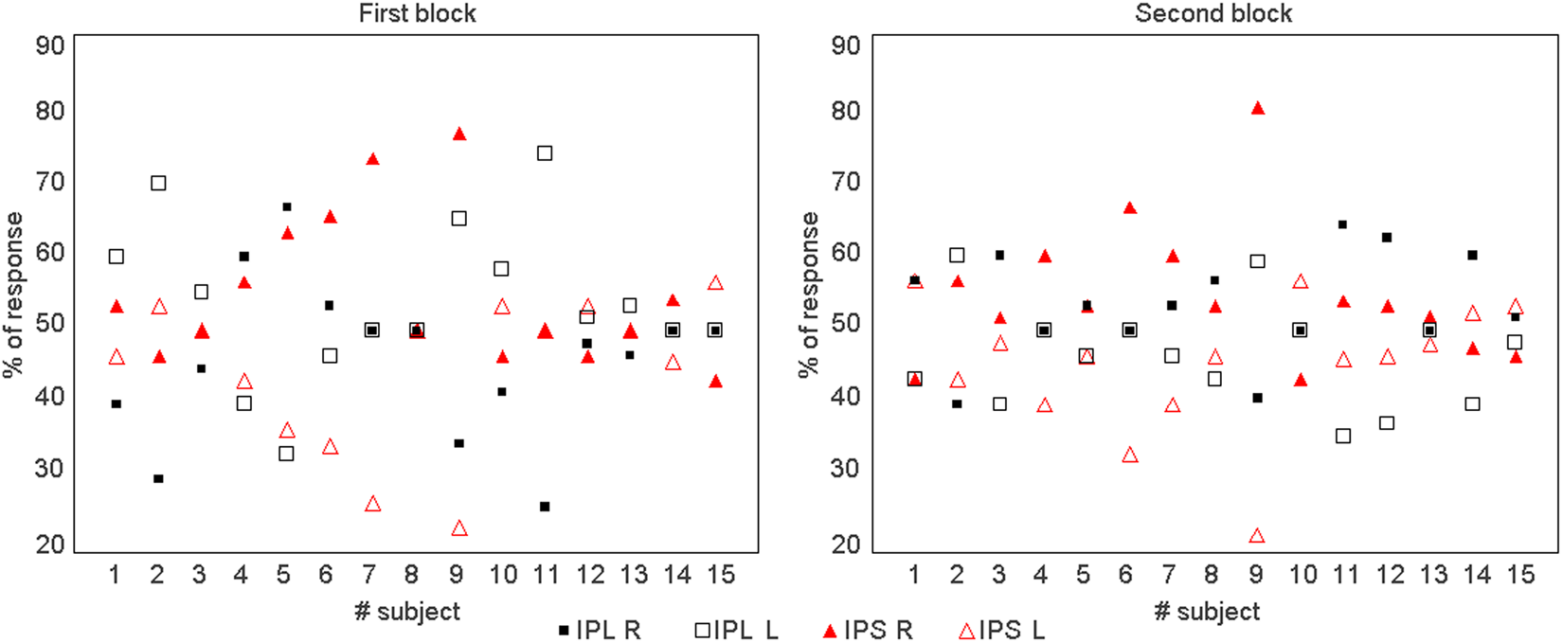
Individual percentage of responses given by the subjects indicating the side (i.e., left or right ear) of the last perceived tone at the end of the tone blocks related to the two stimulation sites (IPL, IPS). For example, IPL R refers to the last tone perceived at the right ear after IPL stimulation.

**Figure 3:**
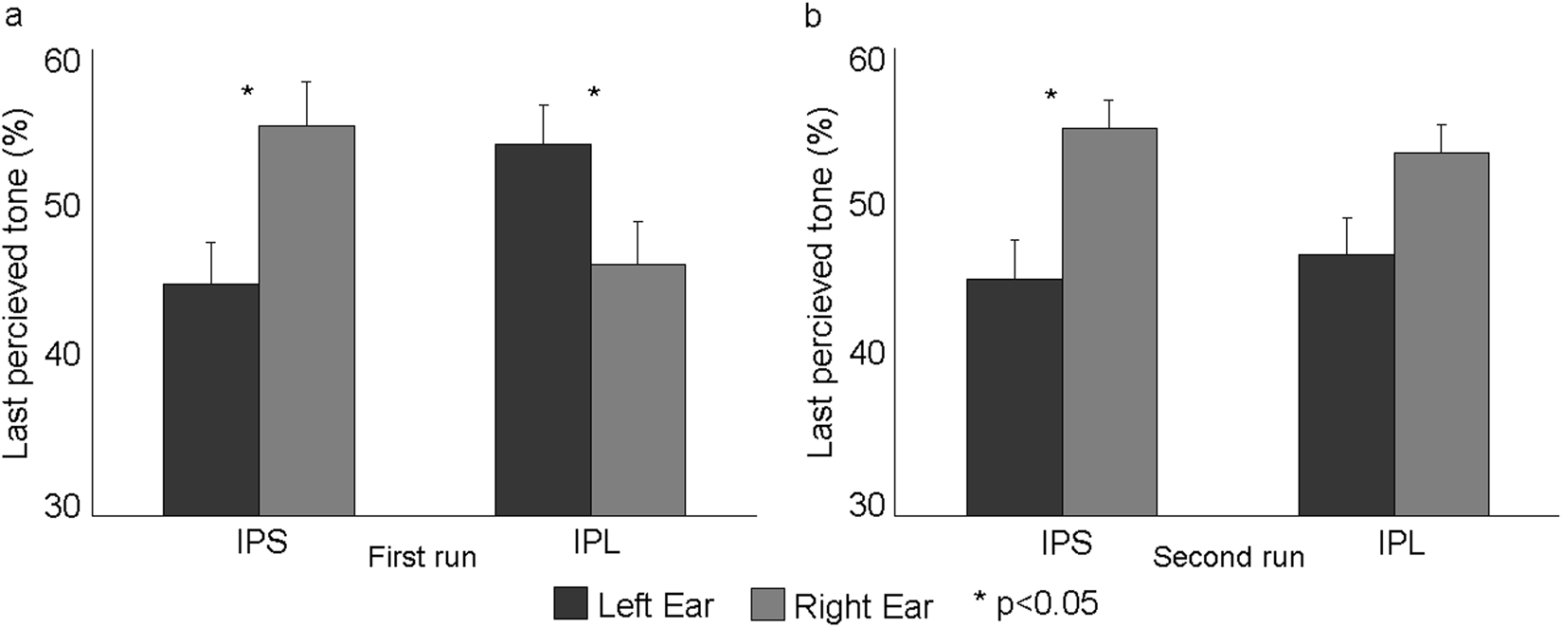
(**a**) Group means (±standard error, SE) of the % of responses given by the subjects indicating the side (i.e., left or right ear) of the last perceived tone at the end of the auditory tone blocks for the two rTMS Conditions (IPS, IPL) as a function of Ear (left and right) in the first run (first 10 minutes). Duncan post-hoc tests: one asterisk (*p* < 0.05). (**b**) Group means (±standard error, SE) of the % of responses given by the subjects indicating the side (i.e., left or right ear) of the last perceived tone at the end of the auditory tone blocks for the two rTMS Conditions as a function of Ear in the second run (second 10 minutes). Duncan post-hoc tests: one asterisk (*p* < 0.05).

Figure 2 shows the individual behavioral results following the two stimulation sites (IPL, pIPS) during the octave illusion in the two blocks, respectively. Specifically, we report the percentage of responses given by the subjects indicating the side (i.e., left or right ear) of the last perceived tone at the end of each block. It can be noted that after the IPS stimulation the majority of the subjects perceived the last tone more frequently in the right ear in both blocks. On the contrary, such pattern was reversed after IPL stimulation in the first (i.e., first 30 auditory stimulations after cTBS) but not in the second block. This observation was confirmed when averaging behavioral results (Fig. 3), where a clear distinct effect was observed in the first run when cTBS was delivered over left IPL or pIPS. This qualitative impression was supported by a significant three-way interaction of Stimulation site (IPL, pIPS), Ear (left, right) and Run (first, second) (F1,14 = 5.24, *p* = 0.038), and relevant Duncan post-hoc tests (*p* < 0.05). In particular, in the first run, after cTBS over the left IPS, subjects on average perceived the last tone more often at the right compared to the left ear (*p* = 0.014). However, after cTBS over the left IPL, the distribution of the side of the last perceived tone was altered, the number of the last perceived tone at the right ear being significantly smaller than at the left ear (*p* = 0.045) (Fig. 3a), thus indicating a modification in the perception of the octave illusion (see also Table 1).

**Table 1:** Percentage of responses given by the subjects at the end of the tone block in the two TMS condition (IPS, IPL) separated by Ear (left, right) and Run (first, second), respectively.

	First run		Second run	
	Left-IPS	Left-IPL	Left-IPS	Left-IPL
Left-ear	44.9 ± 2.6	53.8 ± 2.7	45.2 ± 2.4	46.7 ± 1.8
Right-ear	55.1 ± 2.6	46.2 ± 2.7	54.8 ± 2.4	53.3 ± 1.8

Such a difference was missing in the second run (i.e., second 30 blocks occurring 10 minutes after the end of cTBS). Indeed, following cTBS over both left pIPS (*p* = 0.018) and left IPL (*p* = 0.06), subjects reported the position of the last perceived tone more often at right compared to the left ear, as in the first cTBS session during left pIPS stimulation (Fig. 3b).

The control analysis tested the cTBS effect over the two sites of interest (i.e., left IPL and left IPS) for the two stimuli (i.e., StimA and StimB), separately. Results showed no statistically significant interaction (*p* > 0.5) between TMS Condition (IPL, pIPS), Ear (left, right) and Stim (A, B), thus suggesting that cTBS equally interfered during blocks starting with the different dichotic pair.

## Discussion

We observed a modification in the perception of Deutsch’s octave illusion after magnetic cTBS over left IPL. In particular, such alteration was present in the first run (i.e., first 10 minutes) after cTBS, which was delivered offline to the left IPL before the auditory stimulation. On the contrary, no alteration of the standard illusion (i.e., the last tone more often perceived at the right ear, as observed in the present preliminary experiment and in the referenced study^8^) was noted after cTBS on left pIPS. Later, during the second run (i.e., second 10 minutes), the effect of the magnetic stimulation faded and the perception of the illusion was no longer influenced by the cTBS on left IPL, thus becoming similar in the two brain stimulation conditions. The cTBS effect did not depend on the specific dichotic pair (StimA: 400 left − 800 right; StimB: 800 left − 400 right) and on the ear in which the last tone of the block was perceived.

Overall, our results confirm involvement of the left IPL in the perception of side (illusory ear of origin) during the Deutsch’s octave illusion as previously suggested by the MEG studies of evoked activity^7,8^. Associating brain activity with the subjective report during the illusion, Brancucci and colleagues^8^ found the brain areas specifically processing the illusory percept. In particular, the pitch percept was specifically associated with activity in Heschl’s gyrus, the medial temporal and the superior frontal gyri, as well as in the right inferior frontal gyrus. On the contrary, the side/ear of origin percept was specifically associated with symmetric activity in the left IPL and the superior frontal gyrus. These cortical areas overlap to a good extent the areas that constitute the auditory “what” (pitch) and “where” (ear of origin/side) pathways, respectively. In the past 15 years, these two auditory streams have been found mainly from direct neural recordings in monkeys^16^ and from neuroimaging data in humans^17–20^, following tasks of sound localization. The “what” pathway projects from the anterior primary auditory cortex to more anterior areas, such as the planum polare^21^, the right inferior frontal gyrus and the insulae^22^, and then to the ventrolateral prefrontal cortex^23^. In turn, the “where” pathway projects from the posterior primary auditory cortex dorsally to more medioposterior areas, such as the planum temporale^21^, the IPL and the superior parietal lobe^22^, and then to the dorsolateral prefrontal cortex^23^. Thus, in the present study, the left IPL was confirmed to have a role in spatial aspects of auditory perception since, when its activity was interfered with TMS, the perception of the side or ear of origin of the sound was altered. Thanks to this result, the connection between the role of the left IPL and the resulting behavioral function or perception is assessed more robustly compared to previous neuroimaging results, where the activation of one area or network during a specific behavior does not imply a causal (or strict) role of the area in the observed behavior.

The present results add causal evidence to a series of studies that have been carried out since 1974 aimed at investigating the variables relevant to the physical stimulation and the psychological features of the illusion. In particular, Diana Deutsch proposed a two-channel model to explain the emersion of the octave illusion in terms of separate perceptual “what” and “where” decision mechanisms. Although this explanation is at a psychological level, it anticipates the aforementioned forthcoming evidences on cortical what and where neural auditory pathways. Pertinent to the present research, the “where” mechanism of the two-channel model determines that the percept would be localized in the ear receiving the higher frequency, regardless of which frequency is in fact perceived. Though not directly, the present results substantiate the general structuring of the model in that a specific cortical area, i.e., left IPL, seems to implement one mechanism underlying the illusion, specifically the (deceptive) perception of the side or ear of provenience of the tone.

Considering the promising role of the octave illusion in the study of the NCC^4,5,8^, as a further digression we would like to speculate about the candidacy of the IPL as a cortical area therein involved. Several years ago, Taylor^24^ identified “the inferior parietal lobes as the best candidates for the NCC”. This claim still agrees with a series of convergences that range over neglect, visual extinction, attention, and working memory^25–27^, pointing to a strong implication of the IPL in awareness. Further, the IPL has been found to be involved also in self-awareness^28^ and in the formation of percepts starting from especially complex stimuli^29^. More recently, the parietal lobe has been suggested to be the hub of a posterior *hot zone* which would contain the core of the NCC ^30,31^. This claim is based on the results obtained in experiments designed with no report paradigms which would exclude a primary role of frontal areas in consciousness (as supposed for decades in neuroscience) in favor of more posterior areas. Frontal areas would act to implement the decisions and the motor response requested by the task, and would not be directly involved in consciousness. The use of no report paradigms would be the gold standard to avoid a contamination of the NCC  with such corollary cognitive/motor activity. In this view, the present results on left IPL, although not directly facing the issue of the NCC, constitute a point in favor of a primary role of parietal (and more generally posterior) cortical areas in the generation of perceptual awareness. In fact, the present illusion constitutes an ideal paradigm for the study of the NCCs as it represents one of the few examples of multistable perception in the auditory domain. Each dichotic pair can give rise to 4 possible qualia (i.e. percepts): high tone perceived at the right ear, high tone perceived at the left ear, low tone perceived at the right ear, and low tone perceived at the left ear^3,5^. The implication of IPL found in the present study in the generation of the percept (quale), together with previous fMRI findings^4^ pointing to its activation as a central one during the perception of the illusion, strongly suggests that IPL is an area of the dynamic core of the NCC ^30^ or at least of the neural correlate of auditory consciousness.

As a further speculation, the present results could be interpret in the framework of hemineglect^27^. IPL is part of the parietal lobe, which is known to be a central structure for space perception and codification. The prominent role of the parietal lobe in such a skill is substantiated by a neuropsychological deficit known as spatial hemineglect. Although hemineglect mainly appears after a right parietal lesion resulting in a deficit in contralateral space processing, the present results, obtained after a virtual lesion in the left parietal lobe, could relate to hemineglect. In fact, the increased illusory perception to the left side could be the outcome of a lost imbalance in favor of the right parietal lobe after TMS interference to IPL.

Finally, from a methodological point of view, here we note that cTBS to left IPL modifies the perception of the Deutsch’s octave illusion only in the first run (i.e., 10 minutes) following the stimulation, whereas it was observed that, over different brain regions, cTBS effects are usually more long-lasting (i.e., ~30 minutes)^32^. Nevertheless, it should be considered that the duration of the effects induced by cTBS may itself be affected by the stimulation site, the demands of the task and the physical activity (i.e., hand muscle activity) after the stimulation. For example, cTBS delivered over the dorsolateral prefrontal cortex (DLPFC)^33^ or over the frontal eye field (FEF)^34^ has been shown to produce effects that last for about 15 minutes. According to this point of view, further studies using other cognitive tasks involving different parietal regions will disclose if the present temporally limited cTBS effect is related only to the present task or, more generally, should be ascribed to tasks associated to neural activity of the parietal cortex. Moreover, since to date mostly right-handers have been recruited in neuroimaging studies on the perception of the octave illusion, it would be of interest if future work could disclose whether the left IPL is clearly activated also in a group of left-handed subjects, and, if so, a further TMS study would address its causal role.

## Materials and Methods

### Subjects

Fifteen right-handed volunteers (mean age = 21.3 ± 2.2 years; 9 females), with no previous psychiatric or neurological history, participated in the experiment. Of note, we selected right-handers since both behavioral^1,2^ and neuroimaging studies^11^ have shown a clear pattern of response in the octave illusion only in right-handers. The method of the present study was carried out in accordance with published safety guidelines (see *rTMS procedures and identification of target scalp regions* subsection). All experiments were conducted with the understanding and written informed consent of each participant, according to the Code of Ethics of the World Medical Association, and the standards established by the University of Chieti Institutional Review Board and Ethics Committee. The experimental protocol was approved by the Ethics Committee of “G. d’Annunzio” University of Chieti-Pescara (prot. N° 1558/2017). The experiment was conducted at the Institute for Advanced Biomedical Technologies (ITAB) of the University of Chieti-Pescara. The participants were seated on a comfortable reclining armchair and kept their hands resting on the keyboard of a computer. Subjects were recruited on the basis of their percept in octave illusion as assessed in a preliminary test.

### Stimuli

Sinusoidal 400- and 800-Hz tones (70 dB SPL; rise and fall times 10 ms) with 500-ms duration were synthesized on a personal computer by means of the CSound language^35^. The tones were arranged in a block (Fig. 1a) consisting of the 400- and 800-Hz tones that compose two dichotic pairs (StimA: 400 Hz left, 800 Hz right; StimB: 800 Hz left, 400 Hz right). The pairs were presented alternately without interstimulus intervals; when the right ear received the high tone, the left ear received the low tone and vice versa. The tone blocks, either starting with StimA or StimB, lasted 12 s and were arranged in a pseudorandom sequence comprising 30 blocks with an interval of 8 s (response time + rest) between blocks. This sequence lasted 10 minutes and was presented twice (i.e., first and second experimental run), interleaved by about 3–4 minutes of rest. Hence, the second experimental run started about 13–14 minutes after the stimulation. The acoustic stimulation was provided by headphones connected to the personal computer that also recorded the behavioral data.

During each tone block, subjects were asked to press the space bar on the keyboard to indicate when they started to perceive the alternation between a low-pitch tone at one ear and a high-pitch tone at the other ear, as it happens in the octave illusion. In addition, at the end of each block (i.e., during the time interval of 8 s between two blocks), subjects had to press one of the two buttons describing the subjective properties of the last perceived tone: (1) at the left ear (press “Q” with left index finger), (2) at the right ear (press “P” with the right index finger). Of note, the other illusory aspect of the octave illusion, i.e., tone height, was outside the objectives of the present study and we did not ask for it to avoid contaminations of the response of interest, i.e., tone side. Moreover, we limited our investigation on the “side”, and not the “pitch”, effect of cTBS since magnetic stimulation over frontal areas (i.e., the area involved in the illusory perception of pitch) may be uncomfortable, if not even painful, while it is well tolerated in the parietal regions.

Importantly, the present paradigm minimized possible confounds between attention and consciousness, a central issue in the search of the Neural Correlates of Consciousness (NCC)^36^, because the percept during the octave illusion depends not on attentional shifts of the subject^37^. This also motivated the choice of the left IPS as control site, since this region belongs to the dorsal attention network (DAN), which should not be involved in the illusion perception and is not observed to be activated during our referenced studies^4,7,8^. Furthermore, by stimulating both main (IPL) and control (IPS) sites in the same (left) hemisphere, we avoided the concern that the present results may depend on the fact that the stimulation of IPL merely influences the asymmetry in the brain.

### rTMS procedures and identification of target scalp regions

All participants completed the same task in two sessions: (1) with left IPL stimulation and (2) control session with left pIPS stimulation, session order being counterbalanced across subjects. The two sessions were at least one week apart, scheduled at the same time of day. On both sessions, subjects underwent the same procedure: practice run, continuous theta-burst stimulation (cTBS), and two experimental runs.

TMS was delivered with a focal, figure-eight coil and standard Magstim Rapid 2 stimulator (maximum output 2.2 Tesla). Individual resting motor threshold (rMT) for the left first dorsal interosseous (right motor cortex stimulated) was determined following the standard procedure of Rossini and colleagues^38^. In all experimental sessions, cTBS was delivered to one of the two target sites (left IPL or left pIPS; see Fig. 2b) before the beginning of the task. cTBS consisted of 50-Hz bursts made of three pulses applied with intertrain interval of 200 ms (i.e., at the frequency of 5 Hz). The burst sequence lasted 40 s (amounting to 600 pulses), and the intensity was set at 80% of the individual rMT^39^. Of note, here we preferred to use rMT since it does not depend on the individual (voluntary and barely reproducible across subjects) isometric contraction of FDI muscle, which is necessary for the active motor threshold (AMT). The present parameters are consistent with published safety guidelines^40^ and with previous studies producing an inhibitory effect^33,41^. Of note, it was reported that, when delivered over the primary motor cortex, cTBS induced a decrease in corticospinal excitability for ~20/30 minutes after the end of the stimulation^32^.

The locations of left IPL and left pIPS were automatically identified on the subject’s scalp using the SofTaxic navigator system (E.M.S. Italy, www.emsmedical.net) from a set of digitized skull landmarks (nasion, inion, and two pre-auricular points), about 40 scalp points entered with a Fastrak Polhemus digitizer system (Polhemus), and an averaged stereotaxic MRI atlas brain in Talairach space^42^. The average Talairach coordinates in the SofTaxic navigator system were transformed through a linear transformation to each subject’s scalp. Such a method has a discrepancy of about 5 mm with respect to using individual MRI’s for IPL or pIPS location^43^. This strategy has been successful in previous rTMS studies^44–50^. A mechanical arm maintained the handle of the coil angled at about 45° away from the midline, and the centre of the coil wings was positioned on the scalp to deliver the maximum rTMS intensity over each site (individual peak of activation). The coordinates of the left IPL were selected from a previous MEG study with same experimental paradigm^7,8^ and were as follows: left IPL: −37, −49, 26 (*x*, *y, z* in millimeters). The coordinates of the left pIPS were based on a previous fMRI study assessing task-evoked activity during spatial attention^51^: −25, −67, 48 (Fig. 1b).

### Statistical analyses

Statistical analyses were conducted with within-subject ANOVAs for repeated measures. Mauchley’s test was applied for evaluating the sphericity assumption, Greenhouse–Geisser procedure for correcting the degrees of freedom, and Duncan tests for post-hoc comparisons (*p* < 0.05).

To test the influence of cTBS on the illusion, the percentage of responses given by the subjects at the end of the tone block indicating the last perceived tone at left or right ear was used as the dependent variable, and Stimulation site (IPL, pIPS), Ear (left, right) and Run (first, second) as the within-subject factors.

To test whether cTBS has a similar or different effect on stimulus blocks starting with StimA or StimB, the percentage of responses were used as the dependent variables, and Stimulation site, Ear and Stim as the within-subject factors.

## Data Availability

The dataset generated during the current study is available from the corresponding author on reasonable request.
